# Tissue-Speci**fi**c Quanti**fi**cation of Radiation-Induced Cervical Fibrosis and Correlation with Cervical Range of Motion

**DOI:** 10.21203/rs.3.rs-4516893/v1

**Published:** 2024-06-18

**Authors:** Hendrik Dapper, Maria Waltenberger, Steffi U. Pigorsch, Stephanie E. Combs, Katharina Bauermeister, Wolfgang Bauermeister

**Affiliations:** Technical University of Munich; Technical University of Munich; Technical University of Munich; Technical University of Munich; Technical University of Munich; Kharkiv National Medical University

**Keywords:** Radiation, Fibrosis, Head and Neck Cancer, Shear Wave Elastography

## Abstract

**Background:**

Cervical fibrosis (CF) as a late consequence in patients after radiotherapy significantly impacts the long-term symptoms, functionality, and quality of life of these cancer patients due to a hardening process of different histological tissues. Modern Shear Wave Ultrasound Elastography now enables a differentiated analysis of the changes in various tissue types. In this study, tissue-specific changes in CF induced by radiation therapy in head and neck (ENT) cancer patients were quantified and correlated with cervical range of motion (CROM).

**Materials and Methods:**

16 patients after radiation of the cervical lymphatic drainage were selected as the observation group (OG). Further, 16 people without radiation in the head and neck region were matched by gender, age, and BMI as the control group (CG). Stiffness measurements in kilopascal (kPa; 1 Pa = 1 N m^−2^) were performed using shear wave elastography (SWE) to assess the elasticity of muscle, fascia, and subcutaneous tissue within and surrounding the sternocleidomastoid muscle (SCM). Specific parameters of the OG were compared to the CG and correlated with functional parameters and quality of life (QoL).

**Results:**

The OG exhibited significantly higher stiffness values (Emean, Emax, Emin) across all tissue types than the CG, suggesting a tangible effect of radiation therapy on tissue stiffness. Muscle compartment analysis revealed the most significant stiffness differences. Thickness measurements indicated changes in the muscle and skin but not in the subcutaneous tissue. CROM measurements within the OG fell within normal ranges, suggesting a possible homogenizing effect of radiation treatment on CROM variability. Strong correlations were observed between age and specific stiffness measures, particularly in the OG group, indicating a broader impact of aging or radiation therapy on physiological measures. Significant correlations between tissue stiffness and CROM were found.

**Conclusion:**

CF after radiotherapy occurs primarily in the muscle tissue and its fascia, with the hardening being about twice as pronounced as in the average population and becoming more pronounced with increasing age and correlates with CROM.

## 1 Background

**Figure 1:**
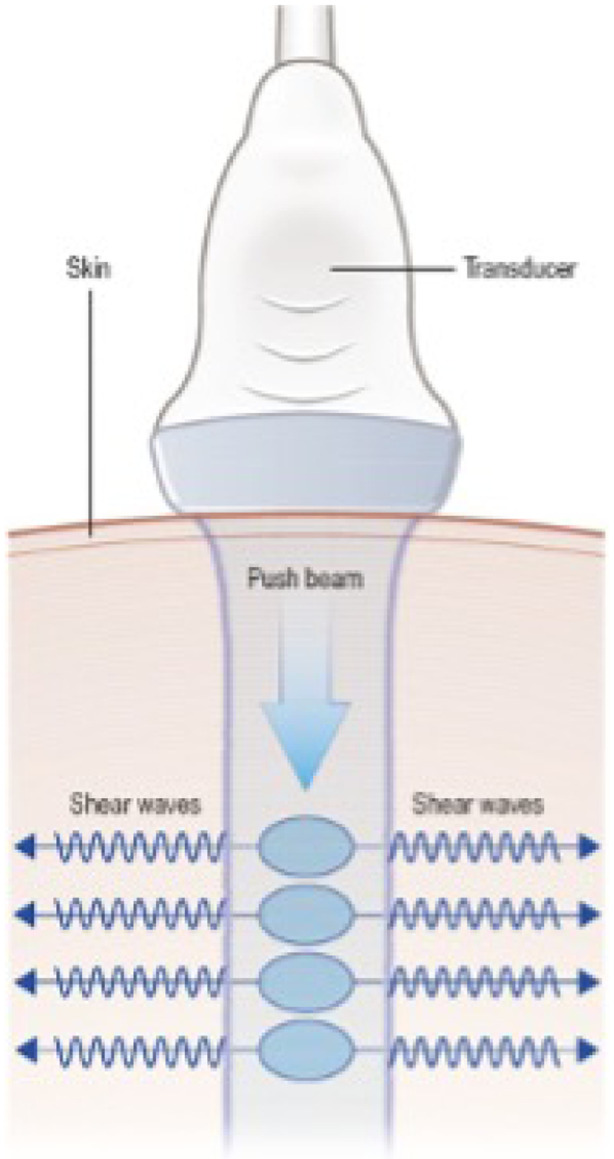
Principle of shear wave elastography: The ultrasound probe emits a push beam, displacing tissue particles. This generates shear waves propagating orthogonally, which are subsequently recorded by the same ultrasound probe.

Fibrosis is a descriptive term that characterizes a dynamic pathological process involving damage to parenchymal cells, stromal tissue remodeling, and the affected tissue’s contraction Straub et al (2015); Stubblefield (2011, 2017); Cox et al (1995). Fibrosis is a late effect of radiotherapy that rarely improves over time and persists as a chronic condition affecting a patient’s functioning and quality of life (QoL) Cox et al (1995); Gupta et al (2021); Andreassen et al (2006). Essentially, fibrosis results from a misdirected wound healing process Mann et al (2011). A crucial component of fibrosis is the hardening of the affected tissue. Blood and lymph vessels get displaced, and fat reservoirs get damaged, which leads to edema, ischemia, and increased vulnerability to injury Straub et al (2015); Koerdt et al (2015); Kosmacek and Oberley-Deegan (2020). As a result, the tissue atrophies and loses its functionality Straub et al (2015); Noel et al (2021). Currently, there is no unified and universally accepted definition of radiation-induced fibrosis. The pathophysiological changes and the resulting symptoms are referred to as “fibrosis”, depending on the context Straub et al (2015); Stubblefield (2011). Therefore, “fibrosis” is often used as an umbrella term for chronic radiogenic changes in various tissues and organs and the resulting symptoms Cox et al (1995). A key reason for this is that it is still largely unclear which specific pathophysiological changes of diverse tissues lead to which symptoms and functional deficits and to what extent these, in turn, affect the QoL. This illustrates the need for more specific consideration of tissue changes and their influence on other clinically relevant consequences, such as functionalities and QoL. One group that is particularly affected by chronic soft tissue changes after radiotherapy is patients with head and neck (ENT) tumors. Due to the close anatomical relationship between various types of healthy tissue and the irradiated tumor, radiation induced cervical fibrosis (RICF) has particular importance for ENT tumor patients. The process of RICF impairs the QoL due to a range of complications, such as cervical dystonia, muscle contracture, dysphagia, and neck muscle atrophy Stubblefield (2011); Kim et al (2015); Nachalon et al (2020); Lindblom et al (2014). With modern intensity modulated radiotherapy (IMRT) Hodapp (2012), significantly lower levels of symptomatic fibrosis can already be achieved compared to conventional 3D radiotherapy. However, approximately 30% of patients still exhibit symptomatic fibrosis of grade 2 or higher one year after radiation Gupta et al (2021). The diagnostic methods for detecting RICF are manifold Stubblefield (2017); Liu et al (2015); Davis et al (2003); Moloney et al (2015). This can be attributed to fibrosis being subject to complex and multiple pathophysiological processes for which no diagnostic standard has yet been defined. The diagnosis generally comprises either the detection of hardening of the tissue or image morphological changes such as induration of the tissue. Historically, however, all these methods have significant limitations. Manual palpation of radiation-induced stiffness in the neck muscles can be classified with a palpation score, but it is subjective Marinus et al (2000). Stiffness as Young’s modulus of elasticity (YM) expressed in kPa through compression with a known force was obtained to validate the palpatory findings. This approach measured the overall stiffness as YM, correlating with the palpation score Leung et al (2002). Another objective approach is indentometry, which measures the overall stiffness in Newton per mm (N/mm) Koch and Wilke (2022). Still, these methods only assess overall stiffness but do not provide any differentiated conclusions about which tissue – subcutaneous tissue, fascia, or muscle – contributes to the overall stiffness. Shear wave elastography (SWE) was first described in 1995 Sarvazyan et al (1995) as a quantitative method for stiffness measurement Liu et al (2015). Shear waves are generated by an acoustic radiation force (Push Beam), causing a displacement of tissue particles, resulting in an orthogonal shear wave generation Bercoff et al (2004); Nightingale et al (2002) (Fig. 1). The ultrasound probe emits the push beam and records the propagated shear waves immediately afterward 1. The propagation speed Cs of the shear wave, measured in m/sec, is higher in stiff and lower in soft tissue. The ultrasound machines can give out both the propagation speed or, through a calculation, the Shear Modulus G (in kPa) or Young’s modulus of elasticity E (in kPa). The expression in kPa is more widely used since clinicians are usually more familiar with the expression of stiffness in kPa. The validity of SWE was examined in a histology-controlled study in a mouse model evaluating skeletal muscle fibrosis, which strongly correlates with histology Martins-Bach et al (2021). Several studies about SWE of muscle tissue have been published for the lower extremities, showing excellent interrater reliability Wu et al (2020). Because the relationships between the extent of connective tissue changes in different tissue types and their clinical relevance of RICF have not yet been sufficiently investigated, we used modern SWE to quantify tissue-specific stiffness in ENT cancer patients without previous surgery in the head and neck area. We compared the results with those of a CG and correlated them with the CROM.

## 2 Methods

### 2.1 Patient selection

From 2020 to 2022, 16 ENT cancer patients were selected as the observation group (OG), and they were treated between 2016 and 2021 with definitive radiochemotherapy, including bilateral, regional lymphatic drainage pathways (at least level IIa to IVb. For inclusion in the study, the completion of the oncological treatment had to be at least 16 weeks previously. The exclusion criteria were operations or functional restrictions in the head-neck region regarding the musculoskeletal system (such as chronic pain and restricted shoulder and neck movement) before radiation. Furthermore, no recurrence and no oncological treatment other than definitive radiochemotherapy had been performed or was pending. Further, 16 patients were included in the study as a CG, and they had not received any radiotherapy in the head and neck region or the shoulder and had never received systemic therapy due to oncological disease. These patients were supposed not to have previous chronic complaints in the head and neck region. The patients in the CG were matched in gender, age, and body mass index.

### 2.2 Clinical baseline assessment

In addition to patient-specific data collection, tumor characteristics, and treatment specifications were collected for all patients. Occurrences of lymphedema and musculoskeletal pain (Numerical rating scale 0–10) were recorded as chronic symptoms. Furthermore, the validated and recognized questionnaires on the general oncological quality of life EORTC QLQ-C30 and the head and neck-specific quality of life of all ENT patients completed HN35. The thickness of the skin, the subcutaneous tissue, and the muscle were measured with the electronic caliper in the offline mode using ultrasound. Additionally, CROM was measured in both groups with the CROM Instrument (Performance Attainment Associates, Roseville, Minnesota, US) with atwo-degree grading (*Appendix Figure A1*). A compass goniometer measured the cervical rotation Audette et al (2010) *(Appendix Figure A2)*. The gravity goniometer measured the cervical lateral flexion. The CROM-Device showed a good inter- and intra-rater-reliability Williams et al (2012).

### 2.3 Ultrasound Examination - Shear Wave Elastography

**Figure 2:**
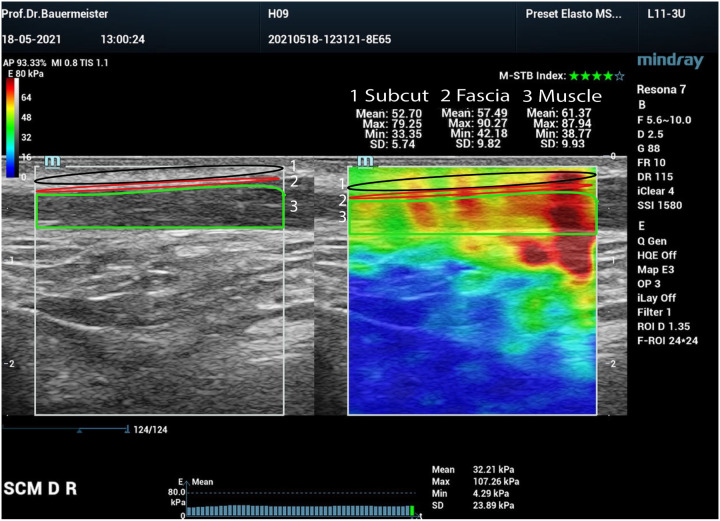
Shear wave elastography of the Sternocleidomastoid muscle (SCM): 1 Trace of the subcuta- neous tissue with E_Mean_ of 52.70 kPa (black frame), 2 Trace of the fascia with E_Mean_ of 57.49k Pa (red frame), 3 Trace of the muscle (green frame) with an EMean of 61.37KPa

The stiffness measurements were done with a Resona 7 (Mindray China) using a linear transducer 11–3 MHz with a width of 43 mm. The subjects were supine with the head resting on a pillow, avoiding stretching on the SCM to avoid a stiffness increase. The entire muscle length was examined left and right in three sections (proximal, middle, and distal). The elastograms were stored for offline analysis. Measurements were obtained using Young’s Modulus E with mean, maximum, and minimum values. The definitive stiffness calculations were done offline using a tracing with an ellipsoid covering the largest possible for the subcutaneous tissue and the fascia and manual tracing for the muscle (Fig. 2). The three measurements of each tissue compartment were averaged to gather information on the entire muscle. The SCM’s transverse diameter was measured by selecting the thickest part of the muscle mass above the bifurcation of the SCM’s sternal and clavicular part.

### 2.4 Statistical Analysis

The analysis was conducted using the Pandas data analysis toolkit. To compare groups, the dependent and independent t-tests were employed for parametric distributions, while the Mann-Whitney U Test was used for non-parametric distributions. Correlation analyses were performed with the Spearman rank correlation coefficient for non-parametric distributions and the Pearson correlation coefficient for parametric distributions.

## 3 Results

### 3.1 Baseline characteristics

A total of 32 patients were included in the study. Of the 16 patients in the OG who received radiochemotherapy for ENT cancer, there were twelve men and four women. The median age was 65; the median BMI was 22,7 kg/m^2^. Most patients had oropharyngeal cancer (9), whereas 4 had hypopharyngeal, two laryngeal and one nasopharyngeal cancer. The median time from the end of treatment to study inclusion was 26 months (range 6–60). 13 of 16 patients had at least one macroscopically suspicious cervical lymph node (cN+) at primary diagnosis. The radiation technique was IMRT for all patients, which was applied by volumetric arc therapy (Varian Medical Systems, Palo Alto,CA, USA) or Tomotherapy (Tomotherapy Hi-Art, Accuray, Sunnyvale, USA). All patients received at least 50 Gy (single dose 2.0 Gy) on elective lymphatic drainage (at least Level II - IV on both sides) and a boost up to 70 Gy (single dose 2.0 Gy) on macroscopic tumor disease. Radiation planning was based on CT and MRI for all patients. Image guided radiation therapy (IGRT) was performed generally and regularly during irradiation. Furthermore, all patients received 4–6 cycles of cisplatin 40mg/m^2^/KOF weekly during radiation therapy. At the time of study inclusion, all patients had a subjectively determined mild degree of fibrosis, which was determined by palpation. 9 out of 16 patients had submental lymph edema, and all nine were currently treated with lymphatic drainage. In the entire course since the end of therapy, 12 patients have ever received lymphatic drainage. Only two patients reported mild musculoskeletal pain in the neck (NRS 2–3/10). 40% of the patients (7/16) stated that they felt tension in the neck and shoulder muscles. The median general score for global health (EORTC QLQ-C30) was 83,3 (mean 75,5; range 41,7–100; SD 15). For the head and neck-specific QoL, EORTC QLQ-HN35, only the answers for pain, swallowing, teeth, opening mouth, dry mouth, sticky saliva, and sense problems were fully completed by all ENT cancer patients. Symptom scales for pain and swallowing were relatively low (median 0 and 8,3) with a small standard deviation (SD 10,0 and 16,7). Half of the patients had no problems with their teeth, while eight patients had values ranging from 33 to 100 (SD of all patients 33.8). Most patients (10) had no problem with mouth opening. However, when it did occur, the extent was pronounced (median 0, SD 42,9). Only five patients had issues with dry mouth (median 0, mean 14,8). The most common limitations were sticky saliva and sensory problems. The most significant restrictions occurred regarding sticky saliva.

Here, 13 out of 16 patients were affected, with a median value of 66.6. Twelve patients (75%) had sensory problems. Twelve patients (75%) had sensory issues, with a mean score of 26.5.

### 3.2 Stiffness - Shear Wave Elastography

The OG exhibited significantly increased stiffness E measured in kPa for the mean, maximum, and minimum measurements in the left and right sternocleidomastoid muscles, fascia, and subcutaneous tissues. This indicates a rise in stiffness across all examined tissue types within the OG, with calculated median ratios from 1.2 to 2.4. Overall, the examination results were very similar on both sides of the neck, which underlines the consistency of the examination method. The hardening of all tissue types in the CG was about half as great as in the OG. Only the median values of the mean and max values in the subcutaneous adipose tissue were only slightly increased compared to the other tissues (ratio 1,2–1,6). In summary of the OG, the mean stiffness values in the muscle and muscle fascia were broadly similar.

In contrast, the stiffness in the subcutaneous fatty tissue was slightly lower (Table 1).

### 3.3 Thickness

#### Cervical Range of Motion

CROM showed no differences between the OG and the CG, with the measurements in both groups falling within the normal ranges. The means and medians of the CROM are presented in Table 3. When considering the scope of movement, an age-dependent differentiation is necessary. The OG showed higher and lower values in specific age and movement categories and a lower incidence of deviations. The OG showed negative correlations with all CROM parameters, indicating a decreased range of motion with age accentuated by RT (Table 4).

### 3.5 Correlations of Stiffness with Function and QoL

Mean Stiffness values of various tissues with the general score for global health (EORTC QLQ-C30), as well as pain, swallowing, opening mouth, and senses problems (EORTC QLQ-HN35), showed very weak or weak mainly negative correlations for all function scores (range: −0,322–0,266). No clear correlations were found for the occurrence of lymphedema either. The values here were between – 0.256 and 0.024.

## 4 Discussion

To our knowledge, this is the first study to quantify RICF in terms of stiffness in a tissue-specific manner. There is a significant hardening of all tissue types compared to the average population, with an approximate doubling of the mean hardening in the muscle and muscle fascia. In contrast, the hardening effects are less pronounced in the subcutaneous fatty tissue. The values can be regarded as very reliable due to the meticulous measurement method and the consistent measurement results on both sides of the neck, which underlines the suitability of SWE for quantifying RICF in soft tissue. From this, it can be assumed that CF follows a relatively reliable inter- individual dynamic. In a previous study by Liu et al, which also investigated muscle stiffness in ENT cancer patients after radiotherapy, the stiffness values E had a normal distribution, whereas, in this study, only E values of the subcutaneous tissue of the right SCM showed a normal distribution Liu et al (2015). The stiffness ratios for the muscle tissue were approximately 3 and 4 for the subcutaneous tissue, which is significantly higher than the ratios for muscle, subcutaneous tissue, and fascia of 2 in our present study. The degree of dispersion is higher in the study by Liu et al. than in the present study when comparing the SD to the MAD multiplied by the factor 1.4826. The differences can be explained by the region of interest (ROI) size, which was significantly larger in our study, resulting in more homogeneous values. Radiation primarily harms tissues by triggering apoptosis or clonogenic cell death through DNA damage caused by free radicals Hauer-Jensen et al (2004). These cause an inflammatory reaction. Increased formation of mediators such as connective tissue growth factor (CTGF) or TGF-*β* leads to stimulation and migration of myofibroblasts and, finally, to increased production of collagen and fibronectin. The connective tissue becomes more rigid and denser, ultimately perceived as hardening Stubblefield (2017); Burger et al (1998); Flechsig et al (2012); Frangogiannis (2020); Rodemann and Bamberg (1995). The complexity of the pathophysiological relationships and their consequences are not fully under- stood. For example, the consequences of radiotherapy regarding the function of the smallest nerve branches have not yet been sufficiently clarified. Peripheral nervous system dysfunction arises from either external compression of soft tissues due to fibrosis, ischemia resulting from fibrosis, or a combination of both factors Stubblefield (2011). We have shown that the resulting hardening effects on the muscle and muscle fascia are relatively stronger than in the subcutaneous fatty tissue. The broadly comparable changes in the muscle and muscle fascia can be attributed to relatively new insights, indicating that fascia tissues are not merely individual supportive structures of a muscle involved in power transmission and proprioception. Instead, they constitute a coherent, multi-layered, and interacting system within the body. They possess complex mechanosensory functions and are even capable of contraction independently Schleip et al (2012); Klingler et al (2014); Schleip et al (2014). The matrix is a dynamic structure with viscoelastic properties and dynamic variabilities. This viscoelasticity can adapt actively to the viscoelasticity of the adjacent tissue by mechanoreceptors Stecco et al (2013). Compared to subcutaneous tissue, the relatively pronounced changes in the muscle and fascia can be explained by increased myofibroblast activity and, thus, the increased incorporation of collagen. On the other hand, a general not fully understood “densification” of the tissue could be responsible, which is indicated by the significant decrease in the tissue thickness of muscle and fascia in contrast to the subcutaneous tissue compared to the CG. Overall, symptoms such as restricted movement or dystonia, pain, or lymphoedema were relatively low in our OG and were essentially evenly distributed. The relationship between radiation dose and volume in the development of radiation-induced fibrosis has already been demonstrated Borger et al (1994). The level of the irradiation dose to the muscle, e.g., correlates with clinical symptoms, as has been shown with the occurrence of trismus in relation to the dose to the pterygoid muscles Goldstein et al (1999); Teguh et al (2008). Both the relatively homogeneous changes in stiffness of all tissues between the respective patients, as well as the moderate musculoskeletal side effects and lymphedema in our OG, can be explained by a very homogeneous dose prescription (50 Gy) of the elective lymphatic drainage pathways with strict adherence to the plan quality using IMRT Hodapp (2012). The essentially good preservation of the mobility of the cervical spine in the irradiated group can also be explained by the widespread strict protection of the myelon and the associated lower dose to the cervical vertebral joints, which is made possible by volumetric arc therapy. Muscle function appears to be largely preserved, despite a general increase in density. Overall, the symptoms in our OG were relatively mild. Only 2 out of 16 patients reported musculoskeletal pain, and the overall quality of life was good. For this reason and because the hardening within the OG was relatively homogeneous and constantly increased, few effects were shown in the correlation analyses with functional parameters and QoL. However, the negative correlations between age and ROM parameters, particularly in the OG, suggest an additive effect of RT and aging on reducing cervical flexibility. This observation is crucial for developing age-specific rehabilitation strategies that account for the compounded impact of RT and natural aging on cervical motion. As described, due to uncertainty and lack of clarity, various radiation-induced effects on different tissue types are grouped as “fibrosis” Straub et al (2015). There is no consensus for a concrete definition or a clear distinction between whether fibrosis is a pathophysiological effect, clinical syndrome, or both. Therefore, the pathophysiological mechanisms of radiation-induced fibrosis must be understood in greater detail, and the explicit causal relationship between these mechanisms and clearly defined clinically relevant symptoms must be elucidated. This is necessary to establish clear dose limits to prevent symptoms and develop targeted potential therapeutic approaches. We have relatively robust dose-volume constraints for many organ functions Teguh et al (2008). However, for musculoskeletal complaints or late effects in soft tissue (such as lymphedema, etc.), there are few correlations between dose exposure and soft tissue complications (lymphedema, tension, pain, restricted movement, etc.). Furthermore, given the complex pathophysiological interplay of musculoskeletal functional impairments after radiotherapy, we still do not know enough about which structures we need to look at precisely when protecting the patient. Dose-volume- related Normal Tissue Complication Probability (NTCP) calculations are necessary for large patient cohorts experiencing higher-grade symptoms to establish meaningful soft tissue constraints regarding higher-grade endpoints. Due to its tissue-specific and reliable, reproducible fibrosis quantification, SWE holds excellent potential for conducting corresponding analyses.

### Limitations.

The OG was relatively small. Additionally, the patients were recruited from regular follow-up care, representing a positive selection bias, as typically mainly healed and generally fitter patients attend follow-up appointments. Both factors contribute to observing mostly mild long-term radiation-induced effects in our OG. There needs to be more than this number of patients to show clear correlations between the various degrees of hardening and quality of life parameters, particularly given the significant inter-individual differences in QoL.

## 5 Conclusion

Cervical fibrosis after radiotherapy occurs primarily in the muscle tissue and its fascia, with the hardening being about twice as pronounced as in the average population and becoming more pronounced with increasing age and correlates with CROM. Shear- wave elastography holds excellent potential for quantifying fibrosis reliably, specifically in various tissue types.

## Figures and Tables

**Table 1:** Stiffness in kPa of the Muscle, Fascia, and Subcutaneous tissue

Measurement	OG Med	CG Med	Ratio	OG MAD	CG MAD	p-value
Mus Mean L	46.98	28.10	1.67	22.29	7.14	0.001
Mus Mean R	46.75	21.52	2.17	16.51	9.55	0.000
Fas Mean L	46.41	25.47	1.82	21.80	8.97	0.002
Fas Mean R	44.28	22.69	1.95	21.61	8.95	0.001
SC Mean L	34.16	23.89	1.43	12.60	6.72	0.001
SC Mean R	34.31	20.92	1.64	10.16	7.05	0.001
Mus Max L	99.87	59.04	1.69	38.60	25.24	0.001
Mus Max R	98.88	47.72	2.07	49.99	23.74	0.000
Fas Max L	67.56	47.35	1.43	31.41	16.68	0.003
Fas Max R	74.24	34.68	2.14	43.52	17.48	0.006
SC Max L	48.57	41.46	1.17	18.17	10.29	0.006
SC Max R	47.54	37.97	1.25	16.88	14.36	0.015
Mus Min L	19.27	12.88	1.50	9.41	4.28	0.014
Mus Min R	20.10	10.06	2.00	7.52	3.40	0.000
Fas Min L	26.94	14.57	1.85	11.76	3.60	0.001
Fas Min R	27.31	14.47	1.89	9.35	3.83	0.001
SC Min L	24.68	13.34	1.85	9.51	5.68	0.000
SC Min R	25.10	14.13	1.78	9.41	5.42	0.001

Fas = Fascia, SC = subcutaneous tissue, L = left, R = right, OG = observation group, CG = control group, Med = Median, Ratio = Ratio of Medians, MAD = Median Absolute Deviation

**Table 2:** Tissue Thickness in mm

Region	Mean OG	Mean CG	sd	p-value
Mus L	7.2	9.8	±2.65	0.000
Mus R	7.8	10.3	±2.27	0.000
Subc L	1.5	1.5	±0.69	0.900
Subc R	1.3	1.7	±0.71	0.090
Skin L	1.5	1.9	±0.38	0.000
Skin R	1.5	2.0	±0.45	0.000

OG = observation group, CG = control group, Subc = subcutaneous tissue, L = left, R = right, sd = standard deviation.

**Table 3:** Cervical range of motion

Measure	Mean/Median* OG	Mean/Median* CG
Rotation L	52.4	57.4
Rotation R	53.7	58.6
Flexion	57.1	51.8
Extension	45.5	43.7
Lat Flexion L	26.0*	26.0*
Lat Flex R	24.5*	24.5*

L = left, R = right.

**Table 4:** Cervical range of motion and correlation coefficients between CROM and Age

Measure	OG Group	CG Group
Rotation L	−0.67	−0.35
Rotation R	−0.66	−0.28
Flexion	−0.49	−0.04
Extension	−0.77	−0.20
Lat Flexion L	−0.64	−0.47
Lat Flex R	−0.64	−0.55

L = left, R = right, OG = observation group, CG = control group.

## Data Availability

All data generated or analyzed during this study are included in this published article.

## Abbreviations

CF: Cervical Fibrosis
CG: Control group
CROM: Cervical range of motion
E: Young’s modulus
EORTC: European Organization for Research and Treatment of Cancer
ENT: Ear, Nose, and Throat
Gy: Gray
SWE: Shear wave elastography
IGRT: Image guided radiotherapy
IMRT: Intensity modulated radiotherapy
OG: Observation group
QLQ: Quality of life questionnaire
QoL: Quality of life
RICF: Radiation Induced Cervical Fibrosis
ROI: Region of interest
RT: Radiation therapy
SCM: Sternocleidomastoid muscle
SWE: Shear wave elastography
